# Engineering biofunctional *in vitro* vessel models using a multilayer bioprinting technique

**DOI:** 10.1038/s41598-018-28715-0

**Published:** 2018-07-11

**Authors:** Jan Schöneberg, Federica De Lorenzi, Benjamin Theek, Andreas Blaeser, Dirk Rommel, Alexander J. C. Kuehne, Fabian Kießling, Horst Fischer

**Affiliations:** 1 Department of Dental Materials and Biomaterials Research, RWTH Aachen University Hospital, Aachen, Germany; 2 Institute for Experimental Molecular Imaging, RWTH Aachen University Hospital, Aachen, Germany; 3 DWI – Leibniz Institute for Interactive Materials, RWTH Aachen University, Aachen, Germany

## Abstract

Recent advances in the field of bioprinting have led to the development of perfusable complex structures. However, most of the existing printed vascular channels lack the composition or key structural and physiological features of natural blood vessels or they make use of more easily printable but less biocompatible hydrogels. Here, we use a drop-on-demand bioprinting technique to generate *in vitro* blood vessel models, consisting of a continuous endothelium imitating the *tunica intima*, an elastic smooth muscle cell layer mimicking the *tunica media*, and a surrounding fibrous and collagenous matrix of fibroblasts mimicking the *tunica adventitia*. These vessel models with a wall thickness of up to 425 µm and a diameter of about 1 mm were dynamically cultivated in fluidic bioreactors for up to three weeks under physiological flow conditions. High cell viability (>83%) after printing and the expression of VE-Cadherin, smooth muscle actin, and collagen IV were observed throughout the cultivation period. It can be concluded that the proposed novel technique is suitable to achieve perfusable vessel models with a biofunctional multilayer wall composition. Such structures hold potential for the creation of more physiologically relevant *in vitro* disease models suitable especially as platforms for the pre-screening of drugs.

## Introduction

The aim of tissue engineering is the replication of functional tissues and organs. The idea of replacing damaged organs or using tissue engineered constructs as novel *in vitro* disease models for basic research and drug testing is driving current three-dimensional (3D) bioprinting research. Since it was proven that the embedment of cells in biocompatible extracellular matrices is associated with a more physiological behaviour^1,2^, there has been an increased effort on the development and use of 3D cell culture and tissue models. A broad variety of tissues has been researched as *in vitro* tissue models including heart, lung, kidney, skin, muscle, and liver^3,4^. According to their physiological relevance and predictive potential, these so-called “organ-on-a-chip” or “tumor-on-a-chip” platforms^5,6^ hold great potential to reduce the number of animal models used by filling the gap between standardized 2D culture assays and necessary *in vivo* experiments. In order to create complex 3D tissue models, different 3D bioprinting techniques and strategies have been successfully established in recent years, and are being continuously refined and improved^7,8^. Nevertheless, the lack of proper nutrient and oxygen supply is still a major constraint for larger constructs. Depending on cellular and matrix composition, the maximum distance for high cell viability is documented to be between 1 and 2 mm^9–11^. For larger tissues with higher complexity 3D bioprinting techniques are used. There are mainly two strategies to create such vascular structures: the direct printing of tubular vessels and the indirect printing of a sacrifice material (e.g. gelatine or Pluronic F127) which is encased in a hydrogel matrix^12^. After hydrogel solidification, the sacrifice material is removed, leaving a hollow tubular structure that can be seeded with endothelial cells^12^. Micro-extrusion-based approaches are currently the preferred choice to print vascular constructs due to their ease of use for generating tubular structures and the processability of a broad range of materials. Vascular channels printed with the extrusion-based printing technique are limited by the size and shape of the nozzle and multiple studies have made use of gels known to provide lower cell responses (i.e. spreading, viability, or migration) but with increased mechanical stability, a feature that is highly relevant if the desired application is a tissue implantation^13–15^. A very promising approach that makes use of native materials such as fibrin or collagen, which are often difficult to handle, was demonstrated by Hinton *et al*. using an extrusion-based system printing into a bath of support material^16^. Microvalve-based bioprinting approaches, on the other hand, only allow the processing of materials with low viscosities such as fibrinogen or gelatine (<5% w/v) but offer higher cell viability and better resolution compared to extrusion based techniques^13,17^. The highest resolutions are achieved by stereolithographic and laser-assisted bioprinting methods. It has been shown, that channels with typical diameters of only a few micrometres to about 125 micrometres could be created using these techniques^18^. To create free standing tubular structures these techniques however did not incorporate cells, while for cellularised systems a patterning technique for photocrosslinkable hydrogels was used or the vessels formed autonomously after a precise deposition^12,18^.

**Figure 1:**
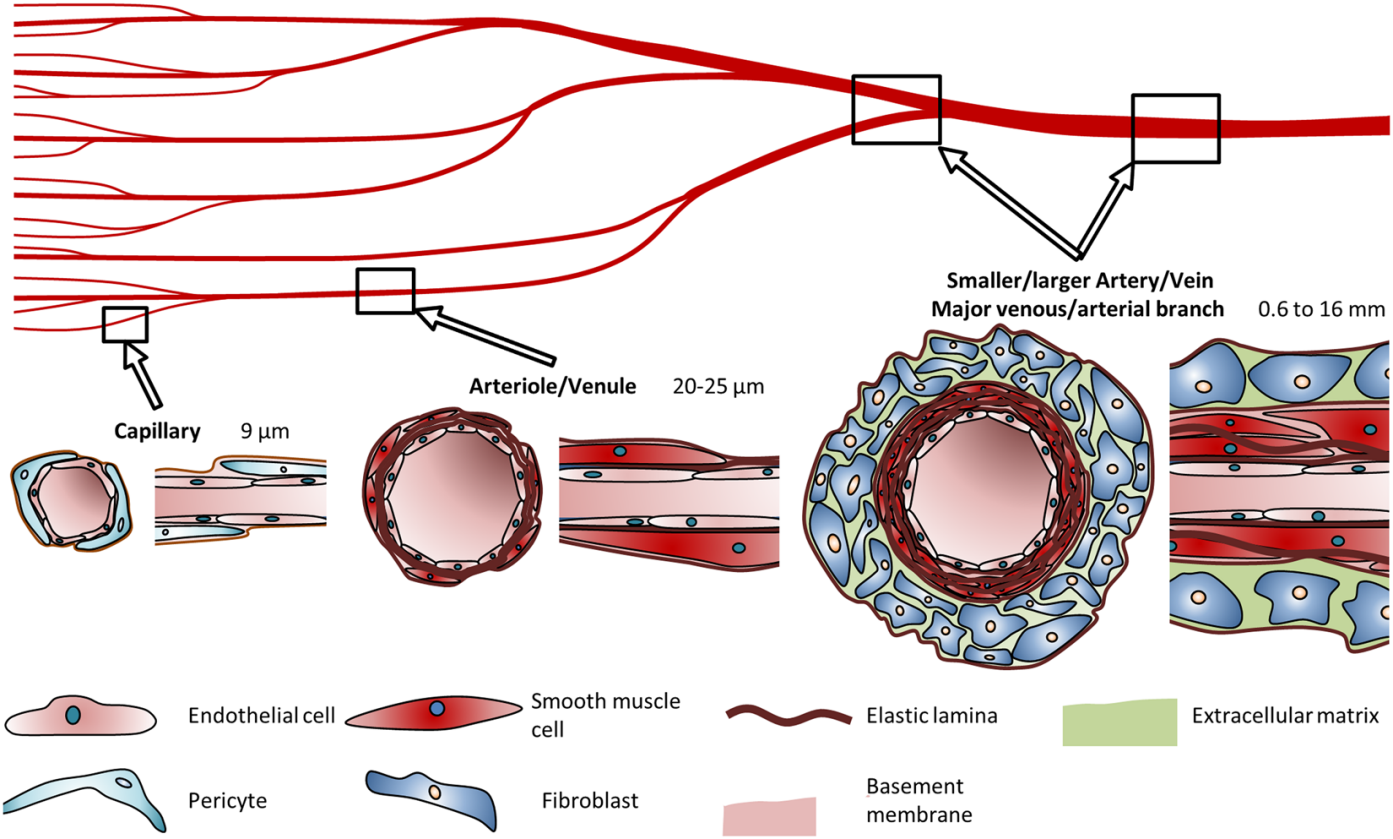
Cell types and composition of vascular channels and their typical diameters. Capillaries comprise a typical EC lining surrounded by pericytes. Arterioles/venules are surrounded by a small number of SMCs. Arteries and veins consist of a dense SMC layer, which is enclosed by fibroblasts.

Recent studies using 3D bioprinting approaches show that it is possible to create thick vascularized tissues (>1 cm) that maintain high cell viability for several weeks^7,10^. The vascular channels spanning the tissue engineered constructs are mostly designed as hollow tubes with a diameter ranging from a few 100 µm^19–21^ up to the millimetre range^9,22^, their inner wall covered by a single layer of endothelial cells (ECs). These printed structures do not fully resemble small arteries and for many applications the complex cellular arrangement of a blood vessel of this particular size may also not be necessary. The purpose of these bioprinted constructs is rather to nourish the cells in the engineered construct or provide increased physical strength due to the materials used. However, if the application for 3D printed vascular structures is primarily the creation of *in vitro* models, either as drug pre-screening platforms or as specific disease models, then the created channels should ideally possess the basic features of natural vessels (i.e. three-dimensionality, endothelium barrier functionality, and maturity) and more closely resemble their native structure in order to achieve meaningful and more predictive results. Therefore, it would be an advantage if the vessel wall would have the cellular arrangement and biofunctionality of a native vessel. For this purpose, one should take into account that there are several types of blood vessels with a different structural composition, depending on the lumen size and physiological function considered. Capillaries, for example, consist of a single layer of ECs, underlying basal lamina, and supporting pericytes. Arterioles as well as venules are supported by additional smooth muscle cells (SMCs) (Fig. 1). Larger vessels in the range of a few 100 µm to the millimetre, however, consist of a single layer of ECs lining the channel, a surrounding layer of smooth muscle cells (SMCs), and an outside layer of fibroblasts and extracellular matrix components (Fig. 1)^23^. The choice of optimised matrices, which should ideally mimic the physiological ECM and ensure proper structural assembly of the construct, is also particularly challenging. The stiffness and mechanical integrity of the matrix need to be carefully considered when it comes to long-term cultivation^24^, while allowing the cells to differentiate, proliferate, and migrate. The stiffness of the material should, therefore, be adapted to the native tissue^25^. Since native materials are often difficult to handle in printing procedures, multiple studies make use of synthetic gels that have been shown to provide low cell responses and high mechanical stability^13,14^. One ideal natural matrix for soft tissues is fibrinogen^26–29^. With the addition of thrombin, the protein crosslinks quickly via a calcium-dependant pathway to fibrin, resulting in a matrix^30^. Due to its strong adhesiveness and good mechanical properties, fibrinogen is widely used as a crosslinking sealant in surgery^31^. Fibrinogen is printable using a submerged extrusion-based printing strategy^16^ and with droplet-based bioprinting techniques^12^. For extrusion-based printing techniques in air it is combined with other hydrogels^14,32,33^, since pure dissolved fibrinogen has a low viscosity and thereby exhibits only low printing accuracy. Another natural hydrogel that plays an important role in vascular structures is collagen^28,34^. It is frequently used in tissue engineering since it is the main component in the extracellular matrix of many tissues^14,35^. However, crosslinking is rather slow^14^ and with the concentrations typically used (<0.4%), collagen hydrogels exhibit poor shape fidelity and consistency due to shrinkage and a low elastic moduli^36,37^.

Here, we present a novel strategy to manufacture vessel models for tissue engineering platforms that resemble the cellular arrangement and responses, as well as the mechanical stability of larger *in vivo* vessels. To achieve this purpose we make use of native hydrogels, in particular fibrin and collagen, with a newly developed multi-layered 3D printing technique. Our technique enables the printing of vascular channels that contain three different cell types in a defined spatial arrangement mimicking native blood vessel configurations. Importantly, we were able to endothelialize our printed blood vessels without the need for the manual injection of endothelial cells after the printing process. With our custom-designed bioreactor, multi-layered models of vascular channels can be dynamically cultivated for longer periods and used for more reliable 3D *in vitro* test systems or disease models.

## Results

### Material characterization

**Figure 2:**
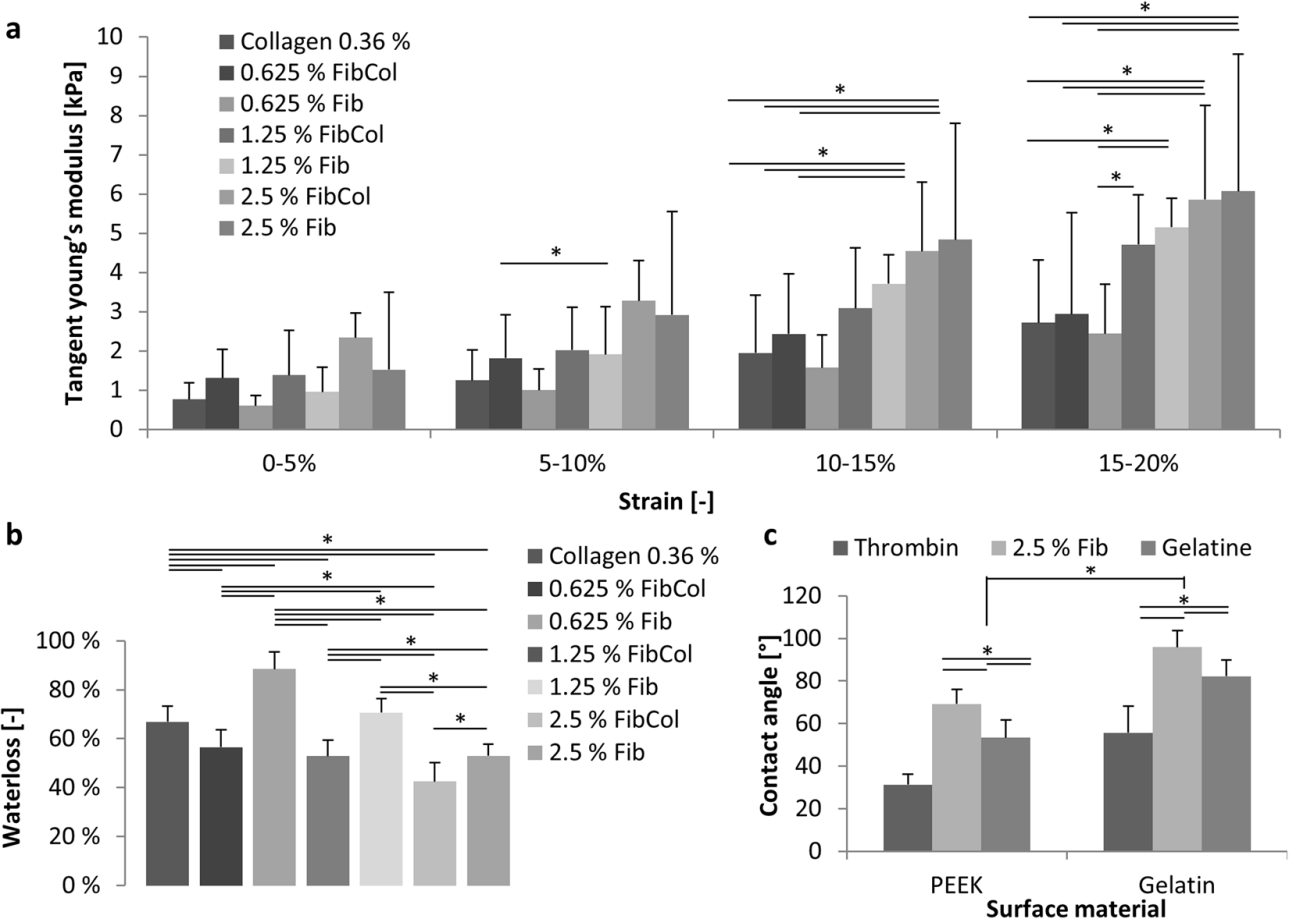
Characteristics of the materials used. (**a**) Tangent Young’s modulus of material combinations used. Measured tangent Young’s modulus between 0 and 20% strain calculated in 5% steps for multiple material combinations (n = 4; *p < 0.05). (**b**) Water loss as a ratio of the original weight after compression. Lower concentrations of fibrin show significantly higher water losses. The addition of collagen reduces the water lost after compression (n = 4). (**c**) Contact angle measurements for thrombin, fibrinogen and gelatine solution on PEEK and gelatine surface materials (n = 6; *p < 0.05).

Material stiffness is considered an important factor in cell behaviour and the compatibility of engineered with *in vivo* tissues^25^. Therefore, we performed compression tests with fibrin and fibrin-collagen blends (Fig. 2(a)) and found that with increasing fibrin content the stiffness of crosslinked hydrogels increased. All materials showed a nonlinear behaviour and a significant increase in stiffness with increasing strain. The stiffness values varied from 0.6 kPa ± 0.2 kPa for the lowest concentration of fibrin, measured between 0 and 5% strain, to 6.1 kPa ± 3.5 kPa for the highest concentration of fibrin (2.5%), measured between 15 and 20% strain. Printed fibrin samples resulted in a compression modulus of 4.2 kPa ± 1.9 kPa for the highest range of strain. At the lower strains, no significant differences in stiffness between different material combinations was observed.

A high loss of water on compression can result in the development of a thin water film between printed constructs and the reactor surface under dynamic cultivation. Over time, these water films can induce a loss of stability and integrity. The water loss on compression (Fig. 2(b)) shows high values for hydrogels containing pure collagen (66.9% ± 6.4%) or pure fibrin with low fibrin contents of 0.625% and 1.25% (88.4% ± 7.1% and 70.7% ± 5.7%). In hydrogels containing 2.5% fibrin, the water loss decreased to 53.0% ± 4.7%. The printed samples resulted in a water loss of 56.4% ± 1.2%, showing no significant differences to their non-printed counterpart. In hydrogel blends containing fibrin and collagen, a significantly lower water loss was observed when compared to the samples without collagen.

The outcome of a drop-on-demand printing process is strongly dependent on the contact angles between the materials used and the surfaces^38^. Larger contact angles typically result in better printability and more defined shapes. In the printing process presented here, the first layer is printed on a PEEK surface, while following layers are mainly printed on the existing gelatine core. We therefore measured the contact angles between the three materials used and the two surfaces. The gelatine surface showed significantly higher contact angles between the liquid and the solid phases than a PEEK surface with all materials (Fig. 2(c)). Fibrinogen (as a 2.5% (w/v) solution) showed the highest contact angles on both surfaces with 69.3 ± 6.8° and 95.9 ± 7.7°. The second highest contact angles resulted from the gelatine solution (5%) with contact angles of 53.4 ± 8.2° and 82.2 ± 7.6° on a PEEK and gelatine surface. Thrombin, as a low viscosity fluid, shows the lowest contact angles of 31.3 ± 5.1° and 55.7 ± 12.6°.

### Cell viability after the printing process

**Figure 3:**
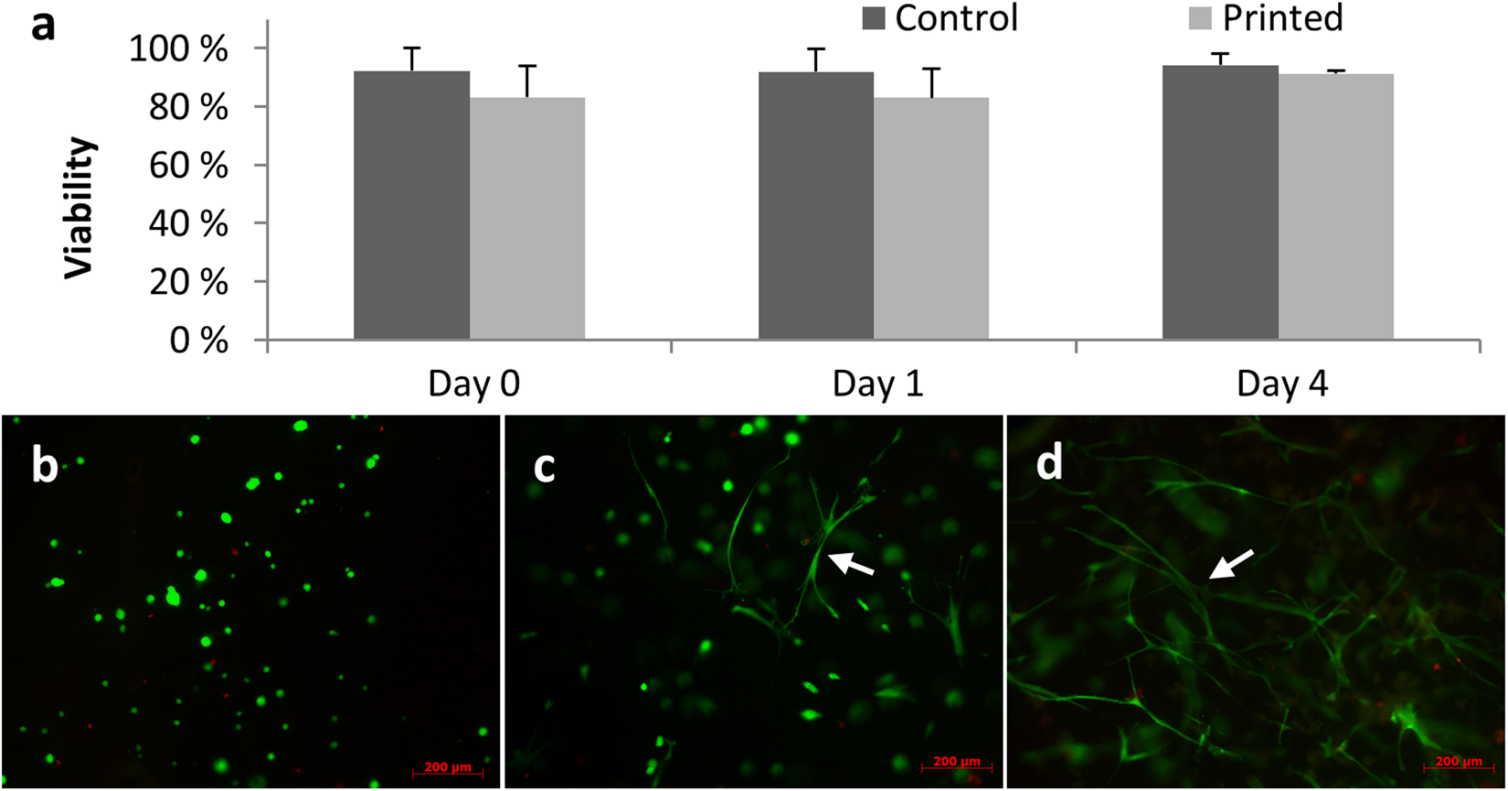
Cell viability after the printing process. Cell viability at day 0, 1 and 4 after preparation comparing control and printed samples (**a**). Cell images on the same day (**b**), after 1 day of cultivation (**c**) and after four days of cultivation (**d**) under fluorescence microscope (Stained with FDA/PI, 10x) showing a round morphology directly after printing and a highly stretched morphology (white arrows) and intercellular contacts for SMCs four days after the printing process.

Based on the above mentioned results, the hydrogel containing 2.5% fibrin appeared most suitable for generating the SMC layer of printed vascular constructs. SMCs printed in these hydrogels showed high survival rates and recovered their stretched morphology within 4 days (Fig. 3). When comparing printed cells to controls that were manually pipetted into the gels, no significant differences in viability were observed (SMC viability, day 0: printed: 83.2% ± 10.9%; pipetted: 92.2% ± 7.9%). At day 4 the viability of printed cells was 91.3% ± 1.1% and of controls 94.2% ± 4.0%. Thus, these data suggest that neither the material nor the printing process is harming the cells in a significant way.

### Vascular channel with a smooth muscle cell layer

**Figure 4:**
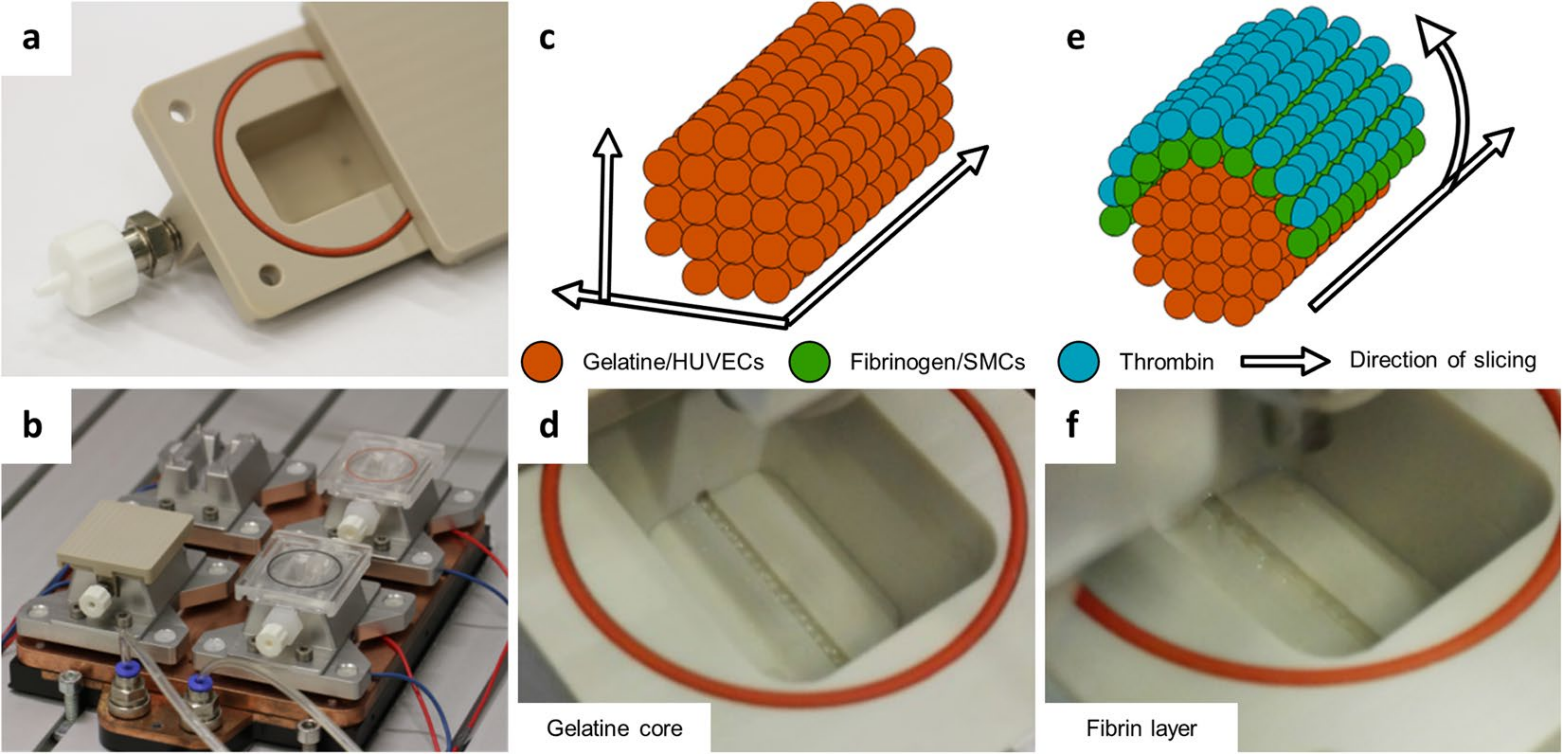
Printing procedure to manufacture vascular channels in custom-made bioreactors. The custom-built bioreactor has Luer lock adapters and an airtight silicon sealing (**a**). The printing process starts with cooling the reactor on the platform for optimal gelation timing (**b**). The gelatine core is printed by following a printing pattern, which is created by slicing the channels horizontally and calculating the droplets in a grid for each longitudinal section (**c**). To print the gelatine core, a single printer head is used at 37 °C (**d**). For the surrounding fibrin layer, the printing pattern is calculated by slicing the channel lengthwise and defining droplet positions in angular steps around the channel outline of each cross section (**e**). Two printer heads are used, filled with fibrinogen and SMCs alternatingly, as well as thrombin as a crosslinker (**f**).

**Figure 5:**
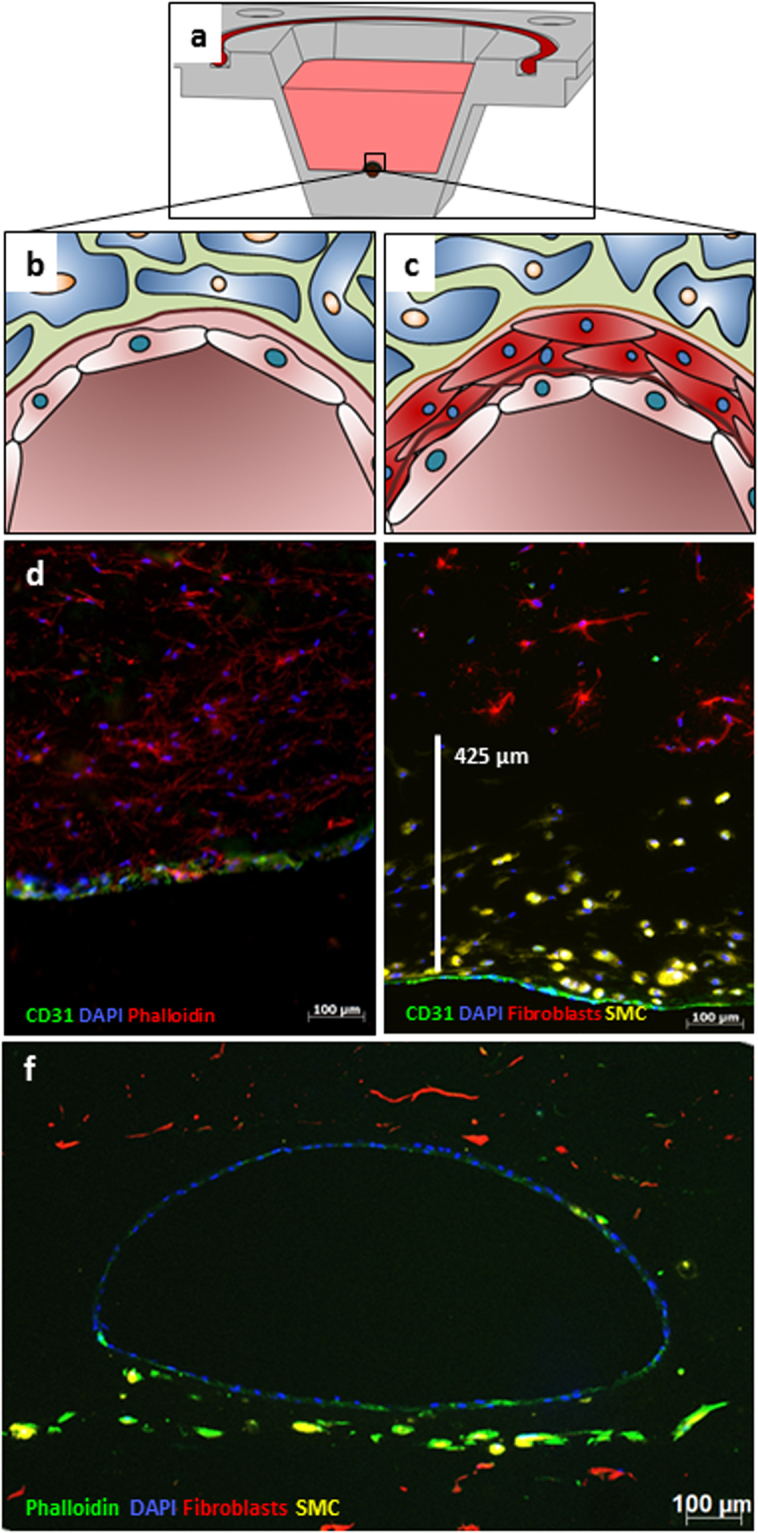
Fluorescence micrographs of vascular-like channels in cross section. Schematic cross section of a reactor shows a single channel (**a**). Schematic cross section of the channel in close-up shows a single layer of endothelial cells (EC) (**b**) and an additional SMC layer (**c**). Confluent layer of EC at the channel wall after 14 days of flow in cross section (**d**) (CD31 in green, Actin-phalloidin in red, DAPI in blue, 10x). Combination of SMC layer and the EC layer showing the distribution of SMCs close to the intact channel as a cross section after 7 days of flow (**e**) (CD31 in green, prelabelled fibroblasts in red, prelabelled SMCs in yellow, DAPI in blue, 20x). A representative fluorescence microscopy image in cross-section highlights the whole architecture of a multi-layered vessel model after 4 days of dynamic cultivation; EC are homogenously distributed in the inner part of the lumen, surrounded by fibroblasts and SMCs. (**f**) (Phalloidin in green, prelabelled fibroblasts in red, prelabelled SMCs in yellow, DAPI in blue, 5x).

A custom-made drop-on-demand 3D bioprinter was used to create the vascular structures in reusable custom-made bioreactors (Fig. 4(a)) positioned on a temperature-controlled platform (Fig. 4(b)). The sacrificial core of the vascular channel was printed with gelatine into 5 °C cold reactors, resulting in a quick gelation of gelatine (Fig. 4(c,d), Supp. Video 1). The optimal distance between two subsequently printed droplets was found to be 0.5 mm. With smaller distances the printing process tends to fuse droplets together, thereby creating an irregular pattern. With greater distances, the single droplets left gaps which led to a regular but incomplete structure. By casting a hydrogel around the sacrifice material, we could prepare simple vascular structures that were lined by a single layer of endothelial cells (Fig. 5(b–d)). Additionally, we were able to print a fibrin layer containing SMC (Fig. 5(c–e), Supp. Fig. 3) on top of the sacrifice layer, before casting the outer hydrogel. The thickness of the SMC layer was up to 425 µm (Fig. 5(d)). However, different wall thicknesses are achievable by adjusting the slicing algorithm (Fig. 4(e,f), Supp. Video 2), the printing pressure, or the opening times of the micro valves (Supp. Fig. 1). When the sacrifice material was removed, our vessel model was 16 mm in length, 1–1.5 mm in width and 0.6–1 mm in height (Fig. 5(f), Supp. Videos 4 and 5).

**Figure 6:**
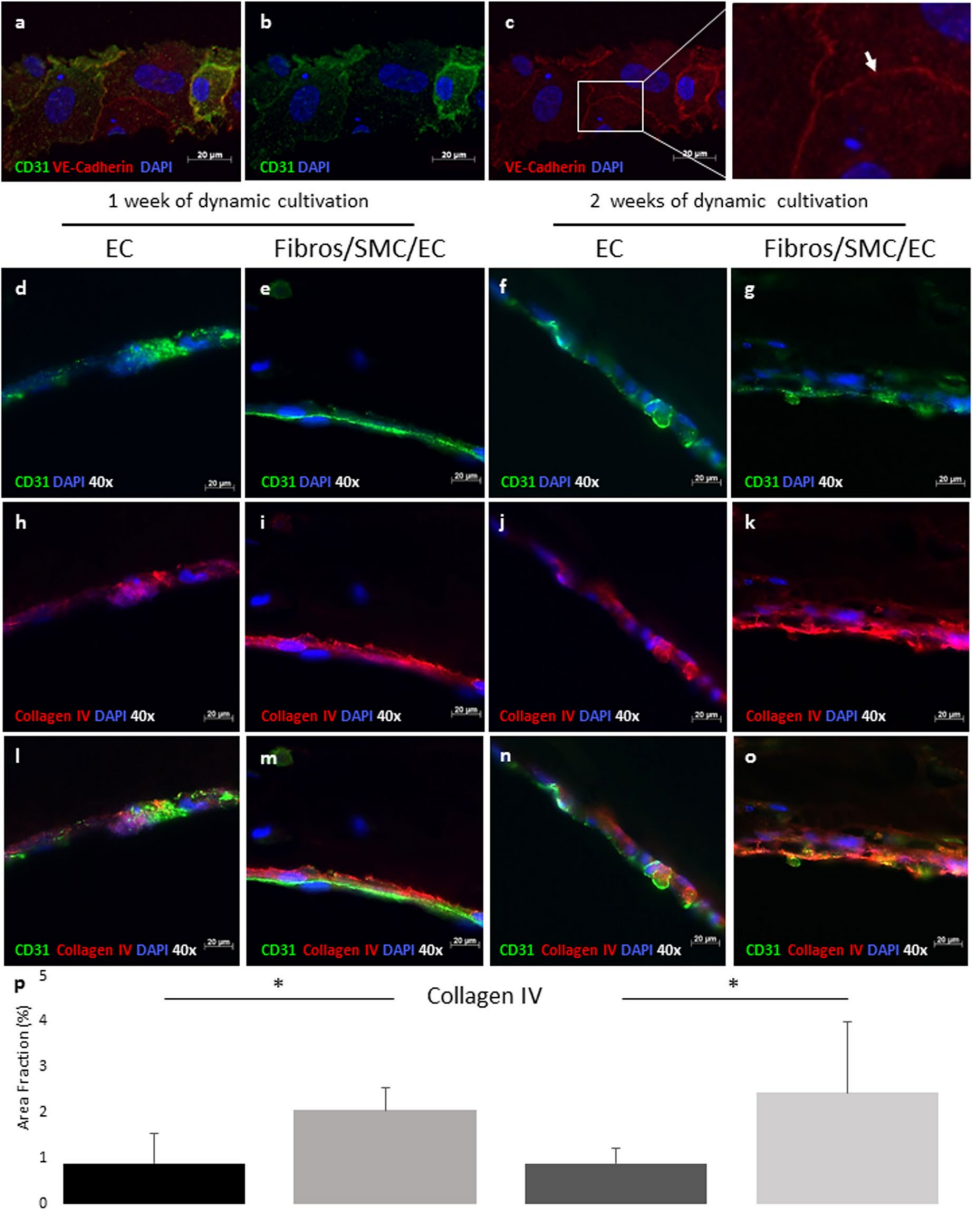
Fluorescence micrographs of the endothelium in sections, qualitative and quantitative assessment of collagen IV expression. Confluent layer of Endothelial cells (EC) at the channel wall after 21 days of flow reveals the expression of VE-Cadherin at the intercellular junctions (white arrow) (**a**–**c**) (CD31 in green, VE-Cadherin in red, DAPI in blue, 40x). Representative fluorescent microscopy images of single channel cross sections reveal increased production of collagen IV after both one and two weeks of dynamic cultivation for the channels lining a single layer of EC (**d**,**f**,**h**,**j**,**l**,**n**) in comparison to the complete multi-layered structures (**e**,**g**,**i**,**k**,**m**,**o**) (CD31 in green, collagen IV in red and DAPI in blue, 40x). A statistically significant increase (*p < 0.05) in the production of collagen IV was also shown by the quantification of collagen IV area fraction (**p**).

**Figure 7:**
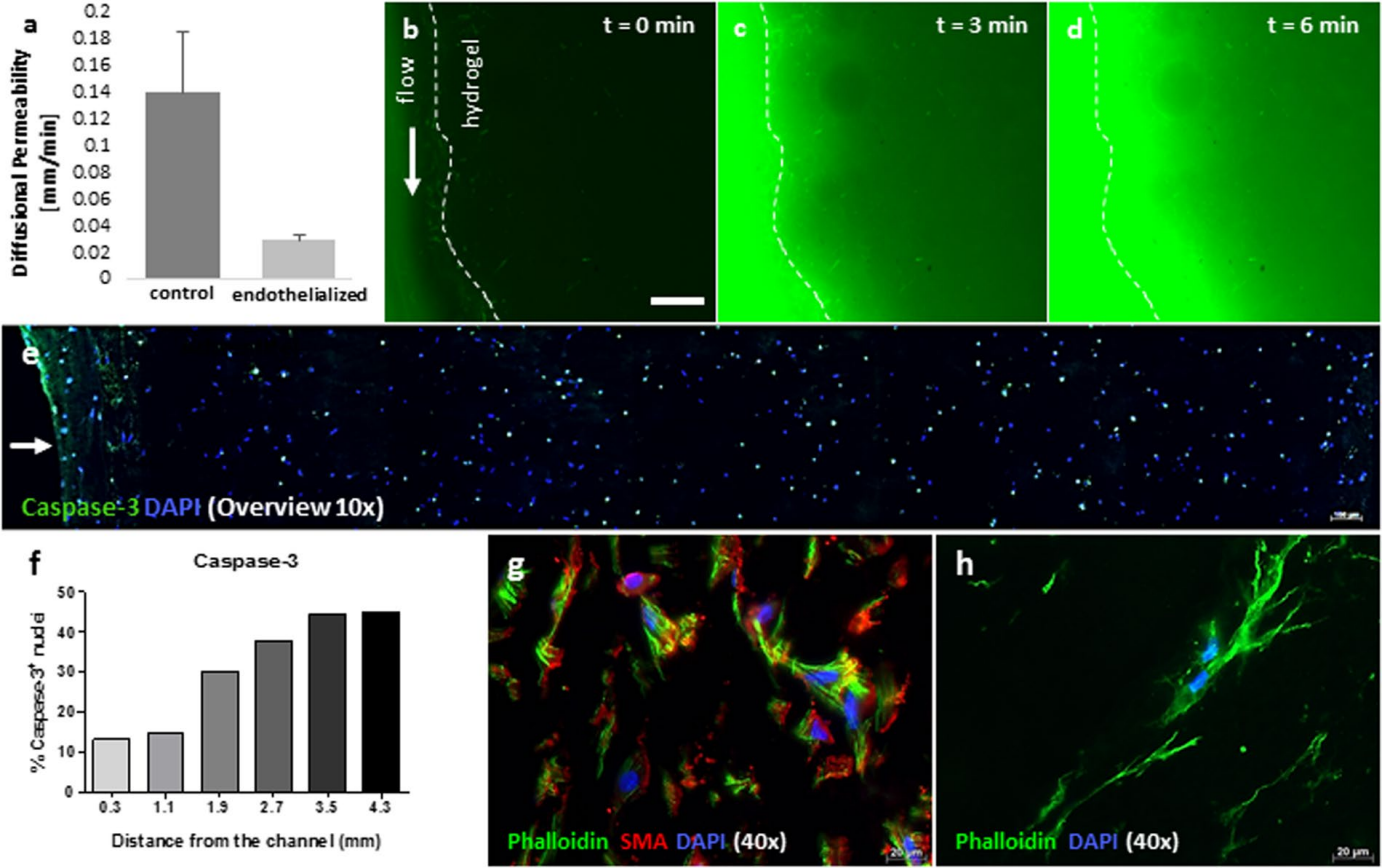
Permeability testing and cell viability evaluation across the construct. In order to assess the endothelial barrier functionality, fluorescent liposomes were added to the medium and perfused through a construct with or without cells. When no cells were present, the diffusional permeability of the liposomes was observed to be almost five times higher compared to the measurement with cells (**a**). Exemplary images of liposome penetration into the hydrogel are shown for t = 0, 3, and 6 min (**b**–**d**). The scale bar represents 500 µm. Cross section overview shows the distribution of caspase-3 positive nuclei along the hydrogel. The position of the channel is marked with a white arrow (**e**) The counting of apoptotic nuclei increases with further distance from the channel (**f**). Phalloidin staining (**g**,**h**) highlights a stretched and elongated morphology of both SMCs and fibroblasts, growing in the 3D hydrogel structure. SMCs are also positively stained for (alpha-) smooth muscle actin (**g**).

The characterization of the HUVEC monolayer revealed the expression of CD31 and VE-cadherin (Fig. 6(a–c)), which play a major role in cell-cell interactions and the maintenance and permeability of a restrictive endothelial barrier^39,40^. To analyse the endothelial functionality and investigate how this is influenced by the multi-layered structure of the *in vitro* vessel model presented here, we examined the expression of collagen IV, primarily found in the basal lamina and known to be essential for the stability of the basement membrane^41^. For this purpose, scaffolds lining either a single layer of endothelial cells or consisting in an additional layer of SMCs surrounded by fibroblasts were dynamically cultivated up to two weeks and analysed for the expression of collagen IV using immunofluorescence-based stainings. These studies revealed that after one week of dynamic cultivation, collagen IV was only visible in proximity to the cell cores in constructs consisting of a single layer of endothelial cells (Fig. 6(d,f,h,j,l,n)). However, when the endothelium was covered by SMCs and fibroblasts, collagen IV levels increased and were detected over the full surface of the channel, showing the typical network structure^42^ after both one and two weeks of dynamic cultivation (Fig. 6(e,g,i,k,m,o)). The quantifications of collagen IV levels show a statistically significant increase in collagen IV expression after both one and two weeks of cultivation for the multi-layered structures in comparison to those consisting of a single layer of EC (Fig. 6(p)). Moreover, an increase in diffusional permeability is measured for non-endothelialized vessels compared to a fully endothelialized vessel after one week of dynamic cultivation (Fig. 7(a–d)).

Cell viability across the construct shows less caspase-3 positive cells in closer distance to the channel (Fig. 7(e,f)). At a distance of roughly 300 µm, only 13.15% of cells are positive for caspase-3 whilst at a distance of 1.9 mm the ratio more than doubles (30.2%). An additional qualitative criterion proving high cell viability is the morphological evaluation of fibroblasts and SMCs. Fibroblasts as well as SMCs show a stretched morphology and the SMCs also show a positive signal for alpha SMA (Fig. 7(g,h)).

## Discussion

Multiple studies have already proven that complex 3D structures are printable and perfusable^10,12,43–45^. While each of these strategies have its target application, a universally applicable system does not exist. For example, some known techniques have made use of materials with a lower cell response for such characteristics as viability, migration, spreading and proliferation, or do not mimic the complex physiological composition of a native vascular channel in relation to their specific size. These might not be relevant if the target application does not require the multicellular design, and the use of physically more stable hydrogels can even be desirable when considered, for example, for implantation. However, cellular responses and biofunctionality are of utmost importance for *in vitro* test systems, e.g. to evaluate drugs or study angiogenesis. For this purpose, a system is required that should ideally resemble the biological composition as well as the mechanical and cellular behaviour of *in vivo* blood vessels. Known methods incorporating smooth muscle cells in close proximity to a channel make use of hydrogels that provide high stiffness but are impaired when it comes to the possibility of providing cell growth and migration^46,47^. We have presented a method to fabricate *in vitro* vessel models using only native hydrogels, which mimics the native composition and spatial cellular arrangement of an *in vivo* blood vessel of the same scale. By comparing the diameters of the designed constructs with native vessels, the *in vitro* channels are comparable to small arteries or veins rather than capillaries or arterioles and venules^48^.

We use a drop-on-demand bioprinting technique with three printer heads working in parallel that makes use of gelatine as a dissolvable core material, thrombin and transglutaminase as crosslinker, and fibrin as well as collagen/fibrin blends as the extra cellular matrix. Our endothelialization technique is based on the idea of mixing HUVECs with the sacrifice material before printing and later, after liquefying the sacrifice material, rather than directly removing the sacrifice material instead waiting two to four hours to allow the endothelial cells to sediment and attach. For the surrounding SMC layer, we used a novel printing strategy to print a fibrin cell suspension as a sealing layer over the core material. This is the first time three different cell types are combined in an automated endothelialization strategy for printed vessel models.

Until now fibrin/fibrinogen has not been not a typical material for 3D bioprinting due to its poor printability, although it provides good gel stability, gelation time, swelling ratio, and cell behaviour for tissue engineered structures and medical sealants^14,31,49^. However, with the presented printing technique we overcome the problematic printability of fibrinogen and make use of the glue-like behaviour of fibrin. This is especially useful for vascular-like structures such as the one presented here in order to provide structural integrity for the dynamically cultivated system^35^.

It was shown that the stiffness of a given tissue also has an influence on the behaviour of growing cells and should ideally be within the range of the target tissue^25,50^. For muscular structures and vasculature the stiffness should be around 10 kPa^51–53^. To mimic the mechanical properties of blood vessels, in our experiments fibrinogen was printed at the highest possible concentration. The stiffness of these samples was not only closest to that of vessels, but they also showed the lowest water loss, which prevents the construct from detaching from the reactor due to the development of a thin water film in between the construct and the reactor. It is noteworthy that the addition of 0.16% collagen to a fibrin sample resulted in less water loss while having no significant effect on stiffness, suggesting that in terms of mechanical properties this combination is favourable. Similar results have been reported elsewhere, showing a lower compaction of the hydrogel mix as well as a higher or similar linear modulus and ultimate tensile stress^29^. Our results suggest that the stiffness could further be increased by increasing the fibrinogen concentration. However, the concentrations used are already near the viscosity limits of inkjet bioprinting^13^. Printed samples did not show significant differences in stiffness when compared to the bulk material, but a trend to lower values could be observed. Yet when comparing the water loss between the bulk material and the printed sample, an insignificantly lower amount of water was lost. In a previous study, a significant decrease in cell viability was observed for materials with increased viscosity and printing pressures, resulting in higher shear rates in the printing process^54^. Therefore, we applied only pressures lower than 0.5 bars, which resulted in high cell viability. Increased valve opening times resulted in significantly larger droplet volumes, offering the possibility to increase the fibrin concentration in the final channel wall. This led to an adjustable ratio of crosslinker solution and fibrinogen by tuning the opening times of the valve. The values show that by using the same opening time and valve size a volume-to-volume ratio between 1.87 and 1.07 of fibrinogen to thrombin solution is achieved. Different final concentrations of fibrinogen can thus be set in a range from 0.53% to 1.83% just by altering the opening times of the valves between 300 and 500 µs. The printing process also allows fine-tuning of the final concentration of fibrinogen by adjusting the number of crosslinker solution droplets printed. However, this could lead to a less homogenous mixing and was therefore not investigated further.

Both endothelialization techniques, the commonly performed injection of HUVECs after dissolving the sacrifice material^9,55^ and the direct printing of HUVECs in the sacrifice material, showed good results for the distribution of cells and their adhesion to the surrounding material. The ECs at the inner surface of the lumen formed a confluent and continuous endothelium after about four days of cultivation. The confluent endothelial layer was functional and impaired the diffusion of fluorescent liposomes into the construct when compared to a non-endothelialized channel. The endothelium matured during the dynamic cultivation of three weeks. Vascular endothelial cadherin (VE-cadherin), which plays a major role in the organization of intercellular tight junctions^39^ and demonstrates a confluent endothelium^56^, was observed and proves the functionality of our vessel models. Additionally, in multi-layered channels under dynamic flow conditions up to two weeks, the typical type IV collagen network^42^ is present and an increase in collagen IV expression was measured in comparison to channels consisting of a single layer of endothelial cells. The structural protein is dispensable during early development, but is known to be fundamental for the maintenance of integrity and the function of basement membranes at later stages and under conditions of increasing mechanical demands^41^.

The use of natural materials ensures high cell compatibility and will enable the generation of more realistic *in vitro* test systems in future. Highly stretched cells were visible within the collagen-fibrin blend as well as the pure fibrin gels, which matches the results of other studies showing excellent cellular response within the gel resulting in network formations of ECs^28,49,57^. Additionally, a relatively low amount of caspase-3 positive cells was found inside of the construct within the first millimetre of the channel. These results suggest that for future work important functional cells should be incorporated around the vessel within a distance of a maximum of one millimetre in order to be supplied with enough nutrients. Our printing experiments showed a suitable distribution of SMCs surrounding the *tunica intima* at a thickness of up to 425 µm with elongated healthy cells, mimicking the *tunica media*. When comparing our results with human *in vivo* small arteries and veins, similar wall thicknesses were observed^58^. However, a reduction in wall thickness is considered a long-term goal in order to be able to more freely design the structure of the resulting vessel wall.

In summary, we investigated a new 3D printing technique to engineer biofunctional *in vitro* vessel models using a drop-on-demand bioprinting device with three printer heads and a custom-built bioreactor. While using (blends of) natural hydrogels for a realistic structural and mechanical model of blood vessels, the model is still bioprintable and maintains the necessary mechanical and biological functionality over the duration of cultivation. The proposed printing technique could be used in the future to produce *in vitro* tumour and angiogenesis models and thereby may reduce the amount of necessary animal models in this field. It could also open new possibilities for the field of engineered vascularized tissue grafts or *in vitro* tissue models due to the resulting native composition of cells and structural integrity.

## Materials and Methods

### Cell culture

To mimic the natural environment of a blood vessel three different primary cell types were used. Human Umbilical Vein Endothelial Cells (EC) (PromoCell, Heidelberg, Germany) and Normal Human Dermal Fibroblasts (PromoCell, Heidelberg, Germany) were cultured under standard cell culture conditions (37 °C and 5% CO_2_) and split when they reached 80% confluence. These primary cells were only used up to passage number 8. Human umbilical artery smooth muscle cells (HUASMC) were isolated from fresh umbilical cords as previously described^59,60^. Briefly, the umbilical arteries were minced into small pieces and distributed on the surface of a cell culture flask for primary explant culture in 10 millilitre of cell culture medium (1x DMEM, GlutaMAX-I + 10% FBS, Gibco). After seven to ten days of cultivation the outgrowth of cells was confirmed by light microscopy (Type 101M, Motic), the artery pieces were removed, and cells were passaged. HUASMCs were isolated from the umbilical cord with informed consent and all experiments and methods were approved and performed in accordance with relevant guidelines and regulations of the Ethics Committee of RWTH Aachen University (EK 218/14). Printed constructs were cultivated with a 1:1 mixture of EC culture medium and fibroblast medium with the addition 0.05% of 0.5% phenol red solution (P0290, Sigma-Aldrich, Germany), and 0.16% of tranexamic acid (Carino, Carinopharm, Elze, Germany). A peristaltic pump (HP, Medorex, Nörten-Hardenberg, Germany) and silicon tubes with an inner diameter of 0.8 mm (Medorex, Nörten-Hardenberg, Germany) were used to dynamically cultivate the constructs within an incubator at 37 °C and 5% CO_2_.

### Hydrogel preparation

Fibrinogen from bovine plasma (F8630, Sigma-Aldrich, Darmstadt, Germany) was dissolved (50 mg/ml) in warm PBS (37 °C) by gently stirring for 7–24 h. A thrombin stock solution was prepared by dissolving thrombin from bovine plasma (100 U/ml) (SRP6555, Sigma-Aldrich, Darmstadt, Germany) in cold (4 °C), sterile PBS. A transglutaminase stock solution was prepared by dissolving microbial transglutaminase powder (60 mg/ml) (SKU: 5060341114533, Special Ingredients, Chesterfield, United Kingdom) in PBS. To prepare a gelatine stock solution, gelatine powder (100 mg/ml) (gelatine type A, G2500, Sigma, St Louis, MO, USA) was dissolved at 70 °C in PBS. Subsequently, the pH was adjusted to 7.4 using 1M NaOH. All solutions were sterile filtered with a 0.2 µm surfactant-free cellulose acetate button filter (SFCA filter, Sigma-Aldrich, Darmstadt, Germany), aliquoted and frozen at −20 °C for later use. Prior to use all materials were unfrozen and kept at 4 °C. Collagen hydrogels were prepared in accordance with the protocol of the manufacturer. Briefly, one part of 0.7 M NaOH and one part of 1 M HEPES-Buffer (L 1613, Biochrom Merck, Berlin, Germany) were mixed together and then combined with the same amount of 10x Dulbecco’s Modified Eagle Medium (4.5 g l–1 D-glucose DMEM, Biochrom Merck, Berlin, Germany). The pH was adjusted between 7.9 and 8.05. To prepare the collagen gel, eight parts of Collagen G (L7213, Biochrom Merck, Berlin, Germany) and two parts of the previously prepared solution were mixed. As final concentrations 2.5%, 1.25% and 0.625% (w/v) of fibrinogen (Fib) were used. For fibrin-collagen blends the fibrinogen solutions were mixed with a constant concentration of 0.18% (v/v) collagen (FibCol). For the sacrificial inner part of the channels, gelatine stock solution was diluted one to one in PBS (5% (w/v) final concentration) and 100x antibiotic antimycotic solution (1% (v/v)) (Sigma-Aldrich, Darmstadt, Germany) was added. The printed crosslinker solution contained thrombin (4 U/ml), CaCl_2_ (0.51 mg/ml) and 100x antibiotic antimycotic solution (1% (v/v)) diluted in sterile PBS.

For the fibrinogen cell material, SMCs were trypsinized, counted, centrifuged, and resuspended (12 million cells/ml) in cell culture medium which was then mixed one to one with a 50 mg/ml solution of fibrinogen containing 1% (v/v) of 100x antibiotic antimycotic solution in PBS. To create the surrounding artificial ECM for each reactor, 0.75 millilitre of collagen was prepared as described above and kept on ice. 2.25 million fibroblasts were trypsinized, counted, centrifuged, and resuspended in 0.375 millilitre of medium. The collagen and the cell suspension were then mixed with 0.375 millilitre of the fibrinogen solution (25 mg/ml), as well as CaCl_2_ (0.51 mg/ml), 100x antibiotic antimycotic solution (1% (v/v), transglutaminase (Meat Glue, Special Ingredients, Chesterfield, United Kingdom) and tranexamic acid (0.16% (v/v)) (Cyklokapron, Pfizer, Karlsruhe, Germany).

### Contact angle measurement

The resulting shape of printed patterns is strongly influenced by the spreading of fluidic droplets on the surface. To assess the printability of the hydrogels used in this study, the contact angles were measured at the liquid-vapour interface between the substances (thrombin (50 U/ml), gelatine (5% w/v), and fibrinogen (2.5% w/v)) on top of the solid phase (gelatine (5% w/v) and PEEK). For the tests, 10 µl were pipetted on top of a flat surface, and the droplet was captured with a macro objective (Canon EF 100 mm 1:2.8 ESM, Canon, Krefeld, Germany). Then, the contact angle was measured using the software ImageJ (Fiji 1.51f, Bethesda, USA).

### Stiffness of hydrogels

To measure their stiffness, the hydrogels were prepared as described above. Agarose (3% (w/v) in H_2_O) was used to create moulds with close to no adhesion towards the cast hydrogels. Therefore, the bottoms of petri dishes with a diameter of 55 mm (VWR, Langenfeld, Germany) were coated with liquid agarose (at 37 °C), which was then cooled down to 4 °C for gelation. Subsequently, moulds with a diameter of roughly 17 mm were cast with agarose using metal cylinders as negatives. The samples were prepared in volumes of 1 millilitre and the printed samples were directly printed into the moulds, resulting in heights of about 3 to 5 mm. All diameters and heights were measured after removing the samples from the mould by cutting the agarose with a scalpel and gently placing the cast test specimens into a petri dish. The specimens were compressed using a universal testing machine (MiniZwick, Zwick, Ulm, Germany) at a cross-head speed of 4 mm/min while measuring the normal force. The stress was calculated by dividing the force by the original specimen area. The strain was given by the current height divided by the original specimen height. The compression was stopped when it reached a maximum load of 200 N.

### Water loss on compression

The water loss of hydrogels was measured by removing the remaining gels from the compression and weighing them. By comparing these values to the weight before compression, the water loss on compression could be calculated using the following equation:

1 $$rati{o}_{waterloss}=\frac{{m}_{uncompressed}-{m}_{compressed}}{{m}_{uncompressed}}$$

### Cell viability after printing

To validate the printing method using living cells in fibrinogen solution, a live/dead staining was performed directly as well as one and four days after printing cells from three different donors. A 2.5% (w/v) fibrinogen solution with 6 million cells per millilitre was printed with a pressure of 0.5 bars and a valve opening time of 450 µs. The material was printed onto sterile glass cover slips in a 24-well plate (5 samples per donor and day). After the printing process, 1 millilitre of medium was dispensed on top of the gels and the cells were incubated at 37 °C and 5% CO_2_. For the control, all samples were pipetted into the well plate. At day 2 after the printing process, the medium was changed. For the live dead staining, 10 μl of fluorescein diacetate (5 mg/ml) (FDA, Sigma-Aldrich, Darmstadt, Germany) in acetone (VWR, Langenfeld, Germany) and 10 μl of propidium iodide (5 mg/ml) (PI, Sigma-Aldrich, Darmstadt, Germany) in Ringer’s solution were mixed with 600 μl Ringer’s solution (B. Braun, Melsungen, Germany). After removing the remaining medium, 20 µl of the staining solution was added to each sample and the samples were directly imaged. Three pictures on different positions in the sample with a 10x magnification and one overview picture with a 5x magnification were obtained per sample and analysed using the software ImageJ (Fiji 1.51f, Bethesda, USA). Living and dead cells were counted in each image individually. The number of living cells divided by the total number of cells (living and dead cells) was used as viability.

### Printing process

Before the actual printing process, the distribution of single droplets in the core structure was calculated using a slicing algorithm, slicing the structure at different heights, and calculating evenly spread droplet positions for the resulting longitudinal sections. However, the deposit positions of droplets for the channel wall were calculated by slicing the channel lengthwise and determining single droplet positions by using an angular calculation along the outlines of the channel cross sections. This technique maintains an even distribution of the SMC layer over the channel surface for all heights. The reactors were kept at 5 °C for the entire printing process by a temperature-controlled platform. At first, the dissolvable core was printed with gelatine (either pure 5% or 3% with additional 10–12 million HUVECs per millilitre) from the first printer head at 37 °C using an electromagnetic micro valve with 0.15 mm diameter. Afterwards, two additional printer heads with 0.3 mm diameters were used to create the channel walls mimicking the *tunica media*. The described crosslinker solution was used in a second printer head and the Fib 2.5% solution containing 6 million SMCs per millilitre was used in a third printer head. The printing pressure was set to 0.5 bars and the valve opening time to 450 µs to achieve a stable droplet volume and a high level of viability^54^. For each calculated droplet, at first one droplet of crosslinker solution followed by one droplet of fibrinogen-cell solution was printed, accomplishing a direct crosslinking within the process that resulted in a thin layer around the core material. The upper part of the presented model containing the surrounding fibroblasts was cast after the printing process was completed using a hydrogel mixture of fibrinogen and collagen (1.25% FibCol), which was quickly crosslinked using thrombin in a final concentration of 8 U/ml. After the printing process, the core material was washed out under laminar flow using warm (37 °C) sterile PBS gently pipetted into the open channel three times (Supp. Video 3). Then, in case no HUVECs were printed in the first step, 1 million HUVECs per construct were suspended in 300 µl of cell culture medium and pipetted through the channels until the medium was visible on the other side of the channel. Afterwards, the channels were closed using silicon tubes at both ends and incubated upside down for about four hours. The remaining liquid and non-attached cells were washed off using culture medium. For the long-term cultivation of up to three weeks, cell culture medium was perfused via silicon tubes using a peristaltic pump at a speed of 0.1 ml/min at 37 °C with a CO_2_ level of 5% and the medium was changed every two to three days. The medium reservoir had air exchange via a 0.2 µm SCFA-button-filter and up to four reactors were perfused in a row at once with a single pump and medium reservoir.

### Lipid bilayer staining

To distinguish between the different cell types within the printed structure, SMCs and fibroblasts were stained before the experiments using two different cell labelling solutions of the Vybrant cell-labelling kit (Thermo Fisher Scientific, Schwerte, Germany). Therefore, for each T-75 cell culture flask 25 µl of the cell-labelling solution was mixed with 5 millilitre of the related medium. Any remaining medium from the cell culture flasks was removed and the cells were incubated with the cell staining medium for 20 minutes. Afterwards the staining solution was removed and cells were incubated with 5 millilitre of fresh medium for 10 minutes. The last step was repeated three times until cells were ready to be trypsinized and used in experiments.

### Permeability test

A permeability test was performed to test the functionality of the endothelial lining. The construct was printed as described above and cultivated for one week before the experiment was performed. Fluorescent liposomes (d = 138 nm), prepared as described elsewhere^61^, were added to the medium reservoir to achieve a concentration of 10 µmol/l. The tube supplying the printed vessel was filled with the liposome-containing medium before the flow was initiated at a flow rate of 200 µl/min. A Leica AF6000 LX microscope was used to acquire images every 3 min for a total duration of 30 min. The diffusional permeability was calculated as described elsewhere^10^.

### Fixation after dynamic culture

After the duration of dynamic cultivations, the vascular constructs were fixed with 4% PFA by completely submerging them and leaving them at 4 °C. After 18 hours, the samples were removed from the reactors, sliced into roughly 5–10 mm thick pieces, which then were placed into plastic boxes doused with TissueTek O.C.T. Compound (Sakura Finetek, Staufen im Breisgau, Germany) and shock frozen with the help of liquid nitrogen. The samples were kept at −80 °C until further use.

### Sectioning, staining and imaging

The frozen samples were sectioned into 10 to 40 µm thick slices and either embedded in Mowiol (Sigma-Aldrich, Darmstadt, Germany) and directly imaged at the microscope or stained using an immunofluorescence-based protocol^62^. To prevent unspecific binding and to permeabilize the cell membrane, the thick hydrogel sections were incubated in 1% BSA (v/v) and 0.1% Triton X-100 (v/v) for one hour at room temperature. Sections which were stained for VE-Cadherin were additionally pre-treated by fixing them with 4% PFA and a heat-mediated antigen retrieval step in citrate buffer at pH 6. All samples were imaged using a fluorescence microscope (Axio Imager.M2, Zeiss, Oberkochen, Germany). After the removal of the BSA/Triton X-100 solution, the samples were incubated with anti-human monoclonal CD31 (RA0259-C.5, ScyTek Laboratories, Logan, USA), collagen IV antibody (ab6311, Abcam, Cambridge, UK), alpha-SMA (ab18147, Abcam, Cambridge, UK), Caspase-3 (ab49822, Abcam, Cambridge, UK) or VE-Cadherin antibodies (D87F2, Cell Signalling Technology, Leiden, Netherlands) for one hour at room temperature and then overnight at 4 °C. To remove the excess of unbound primary antibody, three washing steps with PBS were performed, followed by incubation for two hours at room temperature in a humid chamber with the Alexa 488- (#115-225-166, Dianova, Hamburg, Germany) and Cy3-labelled secondary antibodies (#712-166-153 or #115-165-166 Dianova, Hamburg, Germany), and DAPI. In addition, to stain the cytoskeleton, Promo-Fluor 555 phalloidin (PK-PF555P-7-01, PromoKine, Heidelberg, Germany) was co-administered with the secondary antibodies.

### Confocal laser scanning microscopy

Confocal microscopy measurements were performed using a Leica TCS SP8 setup and employed to assess the 3D vascularized channels. Printed scaffolds were first fixed with 4% PFA (at 4 °C overnight), washed in PBS for several hours, manually cut in approximately 1 mm thick pieces, and then blocked and permeabilized overnight at 4 °C using 1% BSA (v/v) and 0.1% Triton X-100 (v/v). Primary antibodies to CD31 (RA0259-C.5, ScyTek Laboratories, Logan, UT, USA) were incubated with the hydrogel sections for one day in a solution of 0.1% BSA in PBS. Removal of unbound primary antibodies was accomplished using a wash step against PBS for 1 day. Secondary antibodies to CD31, as well as the nuclear counterstain (DAPI) and Phalloidin (Promo-Fluor 488 PK-PF488P-7-01, Promokine, Heidelberg, Germany) were incubated with the hydrogels overnight in a solution of 0.1% BSA (v/v) in PBS and then washed for few hours in PBS before imaging. The samples were deposited on a high precision cover glass (170 µm, No. 1.5H) from Marienfeld. A HC PL APO CS2 20X/0.75 dry objective was used for overview and smaller magnification imaging while a HC PL APO CS2 93X/1.30 glycerol objective with glycerol immersion liquid type G (Refractive index: 1.45) was used for higher magnification imaging. A white light laser source with variable laser wavelength between 470–670 nm was used for excitation of Promo-Fluor 488, DiD and DiL dyes. The Promo-Fluor 488 was excited with ν = 490 nm and the resulting emission was detected at the range of λ = 515–536 nm. The DiL labelled Fibroblasts were excited with λ = 550 nm and the corresponding emission was detected at the range of λ = 565–615 nm. The DiD labelled SMCs were excited with λ = 650 nm and the resulting emission was detected at the range of λ = 660–735 nm. 3D images of the samples were generated by sequential stack-scanning measurements in Z direction.

### Quantification and statistical analysis

For the quantification of collagen IV content, the imaging-processing and analysis software AxioVision (SE64 rel 4.8.3.) was used, while the counting of caspase-3 positive cells was performed with the software Fiji. Statistical analyses were performed using Origin 2017 (OriginLab, Northampton, Massachusetts, USA). Data are presented as means ± standard deviations of the mean (SD). When statistically comparing two groups the Student’s t-test was applied and when comparing more than two groups an analysis of variance (ANOVA) followed by Fisher’s exact test was performed to examine the influence of two independent categorical variables. Results were considered significant for values of p < 0.05.

## Electronic supplementary material

Supporting Information

Supporting Video 1

Supporting Video 2

Supporting Video 3

Supporting Video 4

Supporting Video 5
