# Supplementary material for: Engineering biofunctional in vitro vessel models using a multilayer bioprinting technique
Source: Sci Rep. 2018 Jul 11;8:10430. doi: 10.1038/s41598-018-28715-0 (PMC6041340; doi:10.1038/s41598-018-28715-0)
Supplement: Supplementary file 1 — Supporting Information [file 41598_2018_28715_MOESM1_ESM.pdf]

Engineering biofunctional *in vitro* vessel models using a multilayer bioprinting technique

Jan Schöneberg[1#], Federica De Lorenzi[2#], Benjamin Theek[2], Andreas Blaeser[1], Dirk Rommel[3], Alexander J. C. Kuehne[3], Fabian Kiessling[2] and Horst Fischer[1*]

[1]Department of Dental Materials and Biomaterials Research, RWTH Aachen University Hospital, Aachen, Germany
[2]Institute for Experimental Molecular Imaging, RWTH Aachen University Hospital, Aachen, Germany
[3]DWI – Leibniz Institute for Interactive Materials, RWTH Aachen University, Aachen, Germany
[*]Correspondence should be addressed to H.F. (email: hfischer@ukaachen.de)
[#]Authors contributed equally

Supporting Information

Supporting Methods

Droplet volume measurement

To validate the droplet volume of the different materials depending on the opening time of the printer valves, the material was prepared as described above and loaded into the printer heads. 1.5 ml tubes were weighed before and after printing 1500 droplets of material into the tubes. The measured weight difference was divided by the density to obtain the volume of the single droplets (the density only barely differed). Each test for each condition was performed three times and the mean values and SDs calculated.

By varying the length of time, the valve is open during the printing process, droplet volumes can be adjusted and thereby the ratio of crosslinker to cell solution can be altered. The droplet measurements (Supplementary Fig. 1) show that printing more viscous materials like fibrinogen and gelatine resulted in a (significantly) higher droplet volume. However, the droplet size differed even more, when different valve opening times were used. The highest droplet volumes were measured for fibrinogen (2.5 % and 1.25 % (w/v)) at 500 µs opening time resulting in 0.35 and 0.33 µl per single droplet. By reducing the opening time this value could be adjusted to 0.15 and 0.14 µl at only 300 µs opening time. The droplet volumes increased less for thrombin from 0.13 to 0.20 µl from 300 to 500 µs resulting in overall smaller droplet volumes compared to all other materials at the same opening times.

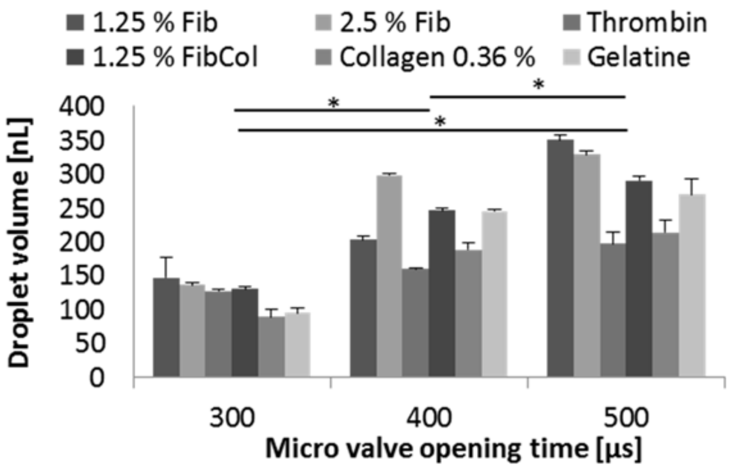

Supporting Figure 1: Droplet volume of a single droplet measured by weight. Different opening times result in significantly different droplet volumes and all material combinations other than collagen to thrombin and gelatine to 1.25% FibCol result in significantly different droplet volumes. Results represent average of 1500 droplets and were repeated three times.

Cell size measurement of endothelial cells

The created HUVEC monolayer inside of the constructed channels builds up after about 4 days of cultivation. To measure the spreading and ongrowth of HUVECs on the inner lumen wall the cell size is was measured directly after fixation samples in PFA overnight. The samples were taken either directly after washing away the gelatine core, or after 1, 2, or 3 weeks of cultivation.

An increase in cell size was only visible comparing the cells directly after washing and covering cells in cultivation, however no difference was significant between 1, 2 or 3 weeks of cultivation, which suggests, that the endothelial monolayer stays intact over the duration of cultivation.

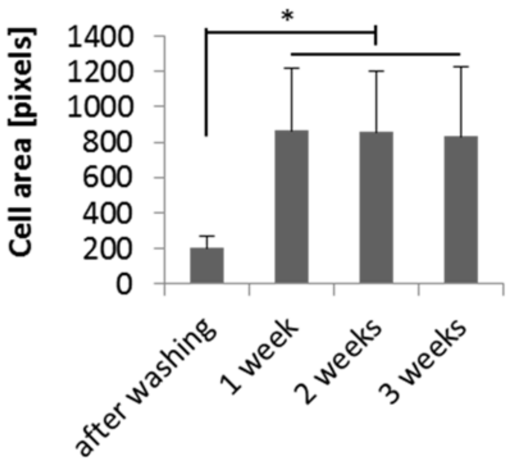

Supporting Figure 2: Size of single cells (gfp-HUVECs) measured in pixels from fluorescence microscopy inside of the channel directly after fixation of PFA. A significant difference was measured between the cell size directly after washing the gelatine out of the channel and the cell size after 1, 2 or 3 weeks of cultivation. No significant change in cell size was visible over the duration of cultivation.

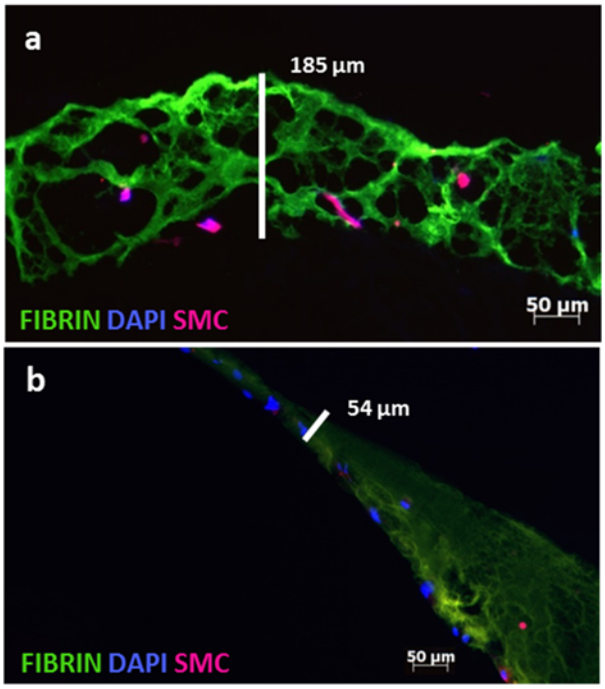

Supporting Figure 3: Size of SMC/fluorescently labelled fibrin layers in a cross-section under the microscopy at different positions and with different layer sizes (Fibrin in green, DAPI in blue and prelabelled SMCs in pink, 10x).

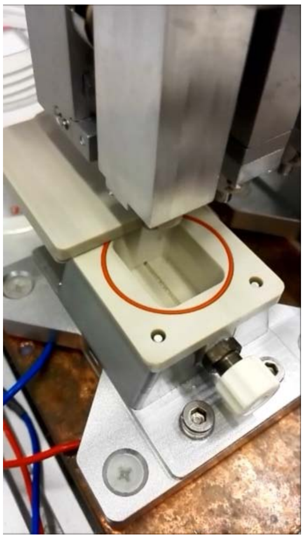

Supporting Video 1: Printing of the gelatine core with a single printer head (2x speed)

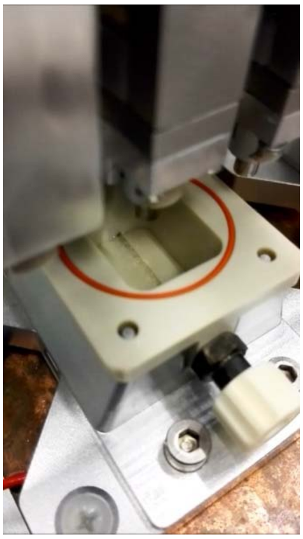

Supporting Video 2: Printing of the fibrin surrounding using two alternating printer heads (2x speed)

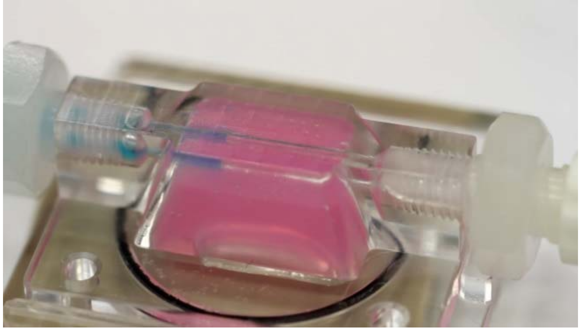

Supporting Video 3: Flushing a vascular channel after printing using blue stained warm (37 °C) water (2x speed)

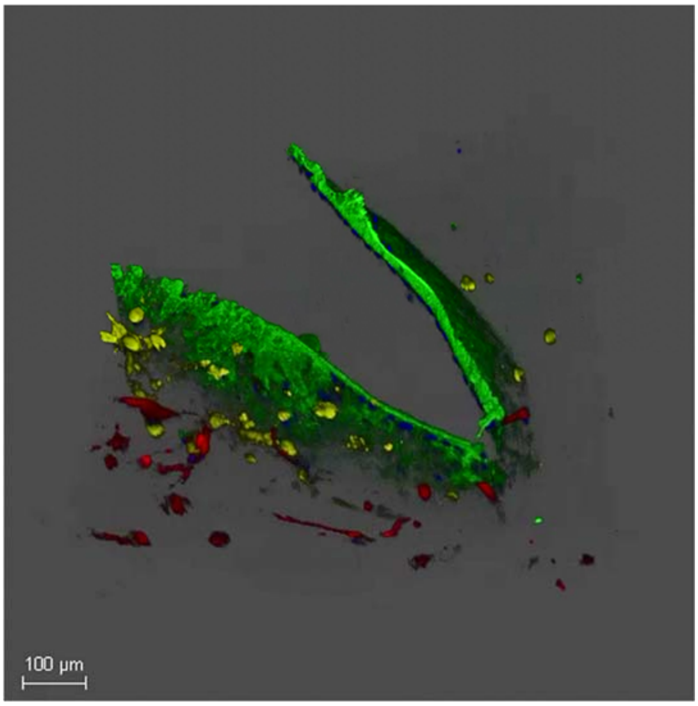

Supporting Video 4: Z-stack of a 200 µm thick slice imaged with the confocal microscope showing the endothelial lining and surrounding cells of a part of the vessel model

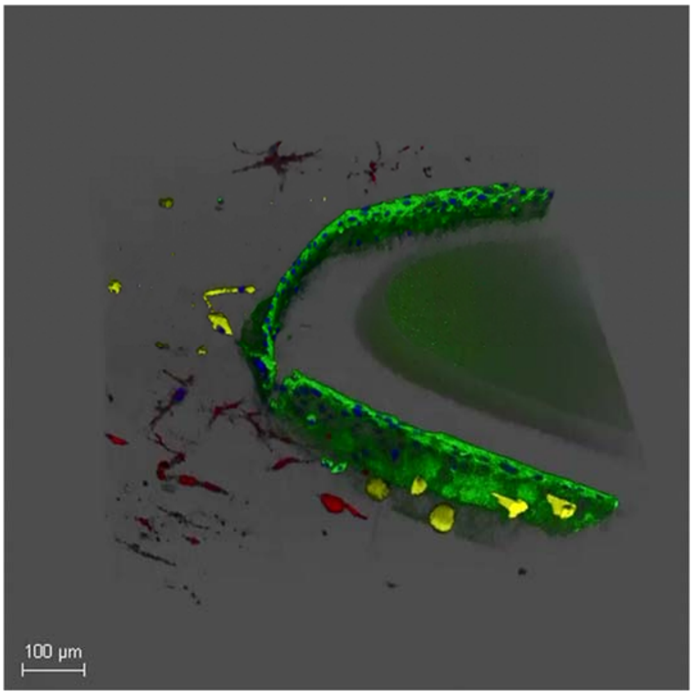

Supporting Video 5: Second Z-stack of a 200 µm thick slice imaged with the confocal microscope showing the endothelial lining and surrounding cells of another part of the vessel model
